# Supplementary material for: Single-cell RNA sequencing and lineage tracing confirm mesenchyme to epithelial transformation (MET) contributes to repair of the endometrium at menstruation
Source: eLife. 2022 Dec 16;11:e77663. doi: 10.7554/eLife.77663 (PMC9873258; doi:10.7554/eLife.77663)
Supplement: Figure 3—source data 1. [file elife-77663-fig3-data1.docx]

**Figure 3 (C)**

| **Column statistics** | **Control** | **24hrs** | **48hrs** | **72hrs** |
| --- | --- | --- | --- | --- |
| Number of values | 21 | 30 | 17 | 6 |
| Minimum | 0 | 9.16 | 1.84 | 0.36 |
| 25% Percentile | 0.175 | 10.58 | 2.455 | 0.6375 |
| Median | 0.38 | 12.57 | 3.62 | 0.96 |
| 75% Percentile | 0.975 | 14.19 | 5.24 | 1.2 |
| Maximum | 1.61 | 18.2 | 5.99 | 1.8 |
| Mean | 0.5362 | 12.78 | 3.712 | 0.9683 |
| Std. Deviation | 0.4588 | 2.496 | 1.369 | 0.4733 |
| Std. Error of Mean | 0.1001 | 0.4558 | 0.332 | 0.1932 |
| Lower 95% CI of mean | 0.3274 | 11.85 | 3.008 | 0.4717 |
| Upper 95% CI of mean | 0.745 | 13.72 | 4.416 | 1.465 |
| Sum | 11.26 | 383.5 | 63.1 | 5.81 |

*One-way ANOVA with Sidak’s multiple comparisons test*

| **Sidak's multiple comparisons test** | **Mean Diff.** | **95.00% CI of diff.** | **Significant?** | **Adjusted P Value** |
| --- | --- | --- | --- | --- |
| Control vs. 24hrs | -12.25 | -13.6 to -10.89 | Yes/**** | <0.0001 |
| Control vs. 48hrs | -3.176 | -4.727 to -1.624 | Yes/**** | <0.0001 |
| Control vs. 72hrs | -0.4321 | -2.634 to 1.77 | No/ns | 0.9957 |
| 24hrs vs. 48hrs | 9.073 | 7.629 to 10.52 | Yes/**** | <0.0001 |
| 24hrs vs. 72hrs | 11.82 | 9.689 to 13.94 | Yes/**** | <0.0001 |
| 48hrs vs. 72hrs | 2.743 | 0.4848 to 5.002 | Yes/** | 0.0094 |
| **Test details** | **Mean 1** | **Mean 2** | **Mean Diff.** | **SE of diff.** |
| Control vs. 24hrs | 0.5362 | 12.78 | -12.25 | 0.4998 |
| Control vs. 48hrs | 0.5362 | 3.712 | -3.176 | 0.5732 |
| Control vs. 72hrs | 0.5362 | 0.9683 | -0.4321 | 0.8132 |
| 24hrs vs. 48hrs | 12.78 | 3.712 | 9.073 | 0.5333 |
| 24hrs vs. 72hrs | 12.78 | 0.9683 | 11.82 | 0.7857 |
| 48hrs vs. 72hrs | 3.712 | 0.9683 | 2.743 | 0.8342 |
