# Supplementary material for: Single-cell RNA sequencing and lineage tracing confirm mesenchyme to epithelial transformation (MET) contributes to repair of the endometrium at menstruation
Source: eLife. 2022 Dec 16;11:e77663. doi: 10.7554/eLife.77663 (PMC9873258; doi:10.7554/eLife.77663)
Supplement: Figure 3—source data 2. [file elife-77663-fig3-data2.docx]

| **Sidak's multiple comparisons test** | **Mean Diff.** | **95.00% CI of diff.** | **Significant?** | **Adjusted P Value** |
| --- | --- | --- | --- | --- |
| Control vs. 24hrs | -12.25 | -13.6 to -10.89 | Yes/**** | <0.0001 |
| Control vs. 48hrs | -3.176 | -4.727 to -1.624 | Yes/**** | <0.0001 |
| Control vs. 72hrs | -0.4321 | -2.634 to 1.77 | No/ns | 0.9957 |
| 24hrs vs. 48hrs | 9.073 | 7.629 to 10.52 | Yes/**** | <0.0001 |
| 24hrs vs. 72hrs | 11.82 | 9.689 to 13.94 | Yes/**** | <0.0001 |
| 48hrs vs. 72hrs | 2.743 | 0.4848 to 5.002 | Yes/** | 0.0094 |
| **Test details** | **Mean 1** | **Mean 2** | **Mean Diff.** | **SE of diff.** |
| Control vs. 24hrs | 0.5362 | 12.78 | -12.25 | 0.4998 |
| Control vs. 48hrs | 0.5362 | 3.712 | -3.176 | 0.5732 |
| Control vs. 72hrs | 0.5362 | 0.9683 | -0.4321 | 0.8132 |
| 24hrs vs. 48hrs | 12.78 | 3.712 | 9.073 | 0.5333 |
| 24hrs vs. 72hrs | 12.78 | 0.9683 | 11.82 | 0.7857 |
| 48hrs vs. 72hrs | 3.712 | 0.9683 | 2.743 | 0.8342 |
