# Supplementary material for: Single-cell RNA sequencing and lineage tracing confirm mesenchyme to epithelial transformation (MET) contributes to repair of the endometrium at menstruation
Source: eLife. 2022 Dec 16;11:e77663. doi: 10.7554/eLife.77663 (PMC9873258; doi:10.7554/eLife.77663)
Supplement: Figure 6—source data 1. [file elife-77663-fig6-data1.docx]

**Figure 6 (C)**

| **Column statistics** | **Control** | **24hrs** | **48hrs** | **72hrs** |
| --- | --- | --- | --- | --- |
| Number of values | 9 | 4 | 9 | 13 |
| Minimum | 0.037 | 15.18 | 0.142 | 0 |
| 25% Percentile | 0.215 | 16.32 | 0.345 | 0.1005 |
| Median | 0.27 | 20.81 | 0.98 | 0.23 |
| 75% Percentile | 0.49 | 22.15 | 1.66 | 0.93 |
| Maximum | 1.77 | 22.24 | 1.77 | 1.6 |
| Mean | 0.4541 | 19.76 | 0.9636 | 0.4824 |
| Std. Deviation | 0.5141 | 3.242 | 0.6402 | 0.5426 |
| Std. Error of Mean | 0.1714 | 1.621 | 0.2134 | 0.1505 |
| Lower 95% CI of mean | 0.05893 | 14.6 | 0.4714 | 0.1545 |
| Upper 95% CI of mean | 0.8493 | 24.92 | 1.456 | 0.8103 |
| Sum | 4.087 | 79.03 | 8.672 | 6.271 |
