# Supplementary material for: Single-cell RNA sequencing and lineage tracing confirm mesenchyme to epithelial transformation (MET) contributes to repair of the endometrium at menstruation
Source: eLife. 2022 Dec 16;11:e77663. doi: 10.7554/eLife.77663 (PMC9873258; doi:10.7554/eLife.77663)
Supplement: Figure 6—source data 2. [file elife-77663-fig6-data2.docx]

*One-way ANOVA with Tukey’s multiple comparisons test*

| **Tukey's multiple comparisons test** | **Mean Diff.** | **95.00% CI of diff.** | **Significant?** | **Adjusted P Value** |
| --- | --- | --- | --- | --- |
| Control vs. 24hrs | -19.3 | -21.17 to -17.44 | Yes/**** | <0.0001 |
| Control vs. 48hrs | -0.5094 | -1.971 to 0.9523 | No/ns | 0.7805 |
| Control vs. 72hrs | -0.02827 | -1.373 to 1.316 | No/ns | >0.9999 |
| 24hrs vs. 48hrs | 18.79 | 16.93 to 20.66 | Yes/**** | <0.0001 |
| 24hrs vs. 72hrs | 19.28 | 17.5 to 21.05 | Yes/**** | <0.0001 |
| 48hrs vs. 72hrs | 0.4812 | -0.8634 to 1.826 | No/ns | 0.7666 |
| **Test details** | **Mean 1** | **Mean 2** | **Mean Diff.** | **SE of diff.** |
| Control vs. 24hrs | 0.4541 | 19.76 | -19.3 | 0.6865 |
| Control vs. 48hrs | 0.4541 | 0.9636 | -0.5094 | 0.5386 |
| Control vs. 72hrs | 0.4541 | 0.4824 | -0.02827 | 0.4954 |
| 24hrs vs. 48hrs | 19.76 | 0.9636 | 18.79 | 0.6865 |
| 24hrs vs. 72hrs | 19.76 | 0.4824 | 19.28 | 0.6532 |
| 48hrs vs. 72hrs | 0.9636 | 0.4824 | 0.4812 | 0.4954 |
