# Supplementary material for: Single-cell RNA sequencing and lineage tracing confirm mesenchyme to epithelial transformation (MET) contributes to repair of the endometrium at menstruation
Source: eLife. 2022 Dec 16;11:e77663. doi: 10.7554/eLife.77663 (PMC9873258; doi:10.7554/eLife.77663)
Supplement: Figure 6—source data 3. [file elife-77663-fig6-data3.docx]

| **Column statistics** | **Control** | **24hrs** | **48hrs** | **72hrs** |
| --- | --- | --- | --- | --- |
| Number of values | 4 | 12 | 6 | 3 |
| Minimum | 0.28 | 0.21 | 0 | 0.38 |
| 25% Percentile | 0.2925 | 0.25 | 0 | 0.38 |
| Median | 0.525 | 0.385 | 0.06 | 0.48 |
| 75% Percentile | 0.93 | 1.083 | 0.355 | 0.99 |
| Maximum | 1 | 1.87 | 0.76 | 0.99 |
| Mean | 0.5825 | 0.6483 | 0.1833 | 0.6167 |
| Std. Deviation | 0.3408 | 0.5568 | 0.2962 | 0.3272 |
| Std. Error of Mean | 0.1704 | 0.1607 | 0.1209 | 0.1889 |
| Lower 95% CI of mean | 0.04018 | 0.2946 | -0.1275 | -0.196 |
| Upper 95% CI of mean | 1.125 | 1.002 | 0.4942 | 1.429 |
| Sum | 2.33 | 7.78 | 1.1 | 1.85 |
