# Supplementary material for: Single-cell RNA sequencing and lineage tracing confirm mesenchyme to epithelial transformation (MET) contributes to repair of the endometrium at menstruation
Source: eLife. 2022 Dec 16;11:e77663. doi: 10.7554/eLife.77663 (PMC9873258; doi:10.7554/eLife.77663)
Supplement: Figure 6—source data 4. [file elife-77663-fig6-data4.docx]

*One-way ANOVA with Tukey’s multiple comparisons test*

| **Tukey's multiple comparisons test** | **Mean Diff.** | **95.00% CI of diff.** | **Significant?** | **Adjusted P Value** |
| --- | --- | --- | --- | --- |
| Control vs. 24hrs | -0.06583 | -0.8034 to 0.6717 | No/ns | 0.9944 |
| Control vs. 48hrs | 0.3992 | -0.4254 to 1.224 | No/ns | 0.5434 |
| Control vs. 72hrs | -0.03417 | -1.01 to 0.9415 | No/ns | 0.9997 |
| 24hrs vs. 48hrs | 0.465 | -0.1737 to 1.104 | No/ns | 0.2093 |
| 24hrs vs. 72hrs | 0.03167 | -0.7929 to 0.8563 | No/ns | 0.9995 |
| 48hrs vs. 72hrs | -0.4333 | -1.337 to 0.47 | No/ns | 0.5506 |
| **Test details** | **Mean 1** | **Mean 2** | **Mean Diff.** | **SE of diff.** |
| Control vs. 24hrs | 0.5825 | 0.6483 | -0.06583 | 0.2646 |
| Control vs. 48hrs | 0.5825 | 0.1833 | 0.3992 | 0.2958 |
| Control vs. 72hrs | 0.5825 | 0.6167 | -0.03417 | 0.35 |
| 24hrs vs. 48hrs | 0.6483 | 0.1833 | 0.465 | 0.2292 |
| 24hrs vs. 72hrs | 0.6483 | 0.6167 | 0.03167 | 0.2958 |
| 48hrs vs. 72hrs | 0.1833 | 0.6167 | -0.4333 | 0.3241 |
