# Supplementary material for: Single-cell RNA sequencing and lineage tracing confirm mesenchyme to epithelial transformation (MET) contributes to repair of the endometrium at menstruation
Source: eLife. 2022 Dec 16;11:e77663. doi: 10.7554/eLife.77663 (PMC9873258; doi:10.7554/eLife.77663)
Supplement: Figure 7—source data 1. [file elife-77663-fig7-data1.docx]

| **Column statistics** | **Control** | **24hrs** | **48hrs** | **72hrs** |
| --- | --- | --- | --- | --- |
| Number of values | 9 | 4 | 9 | 13 |
| Minimum | 0.23 | 2.04 | 12.4 | 10.3 |
| 25% Percentile | 0.53 | 2.105 | 15.1 | 11.09 |
| Median | 0.69 | 3.71 | 17.2 | 12.55 |
| 75% Percentile | 0.985 | 5.383 | 18.31 | 15.33 |
| Maximum | 1.46 | 5.47 | 19.8 | 17.9 |
| Mean | 0.75 | 3.733 | 16.63 | 13.26 |
| Std. Deviation | 0.3583 | 1.813 | 2.248 | 2.421 |
| Std. Error of Mean | 0.1194 | 0.9065 | 0.7492 | 0.6715 |
| Lower 95% CI of mean | 0.4746 | 0.8476 | 14.91 | 11.8 |
| Upper 95% CI of mean | 1.025 | 6.617 | 18.36 | 14.73 |
| Sum | 6.75 | 14.93 | 149.7 | 172.4 |
