# Supplementary material for: Single-cell RNA sequencing and lineage tracing confirm mesenchyme to epithelial transformation (MET) contributes to repair of the endometrium at menstruation
Source: eLife. 2022 Dec 16;11:e77663. doi: 10.7554/eLife.77663 (PMC9873258; doi:10.7554/eLife.77663)
Supplement: Figure 7—source data 2. [file elife-77663-fig7-data2.docx]

*One-way ANOVA with Tukey’s multiple comparisons test*

| **Tukey's multiple comparisons test** | **Mean Diff.** | **95.00% CI of diff.** | **Significant?** | **Adjusted P Value** |
| --- | --- | --- | --- | --- |
| Control vs. 24hrs | -2.983 | -6.213 to 0.2483 | No/ns | 0.0788 |
| Control vs. 48hrs | -15.88 | -18.42 to -13.35 | Yes/**** | <0.0001 |
| Control vs. 72hrs | -12.51 | -14.84 to -10.18 | Yes/**** | <0.0001 |
| 24hrs vs. 48hrs | -12.9 | -16.13 to -9.67 | Yes/**** | <0.0001 |
| 24hrs vs. 72hrs | -9.53 | -12.6 to -6.456 | Yes/**** | <0.0001 |
| 48hrs vs. 72hrs | 3.371 | 1.04 to 5.702 | Yes/** | 0.0024 |
| **Test details** | **Mean 1** | **Mean 2** | **Mean Diff.** | **SE of diff.** |
| Control vs. 24hrs | 0.75 | 3.733 | -2.983 | 1.19 |
| Control vs. 48hrs | 0.75 | 16.63 | -15.88 | 0.9338 |
| Control vs. 72hrs | 0.75 | 13.26 | -12.51 | 0.859 |
| 24hrs vs. 48hrs | 3.733 | 16.63 | -12.9 | 1.19 |
| 24hrs vs. 72hrs | 3.733 | 13.26 | -9.53 | 1.133 |
| 48hrs vs. 72hrs | 16.63 | 13.26 | 3.371 | 0.859 |
