# Supplementary material for: Single-cell RNA sequencing and lineage tracing confirm mesenchyme to epithelial transformation (MET) contributes to repair of the endometrium at menstruation
Source: eLife. 2022 Dec 16;11:e77663. doi: 10.7554/eLife.77663 (PMC9873258; doi:10.7554/eLife.77663)
Supplement: Figure 7—figure supplement 1—source data 1. [file elife-77663-fig7-figsupp1-data1.docx]

| **Sidak's multiple comparisons test** | **Mean Diff.** | **95.00% CI of diff.** | **Significant?** | **Adjusted P Value** |
| --- | --- | --- | --- | --- |
| iPdgfra-CreERT2;Rosa26-tdTm - iNG2-CreERTM;Rosa26-tdTm | | | | |
| Control | 0.2786 | -1.132 to 1.689 | No/ns | 0.978 |
| 24hrs | 3.269 | 1.241 to 5.298 | Yes/*** | 0.0004 |
| 48hrs | 16.21 | 14.35 to 18.06 | Yes/**** | <0.0001 |
| 72hrs | 12.9 | 10.65 to 15.15 | Yes/**** | <0.0001 |
| **Test details** | **Mean 1** | **Mean 2** | **Mean Diff.** | **SE of diff.** |
| iPdgfra-CreERT2;Rosa26-tdTm - iNG2-CreERTM;Rosa26-tdTm | | | | |
| Control | 0.75 | 0.4715 | 0.2786 | 0.5511 |
| 24hrs | 3.733 | 0.4631 | 3.269 | 0.7927 |
| 48hrs | 16.63 | 0.4283 | 16.21 | 0.7236 |
| 72hrs | 13.26 | 0.3633 | 12.9 | 0.8794 |
