# Supplementary material for: Single-cell RNA sequencing and lineage tracing confirm mesenchyme to epithelial transformation (MET) contributes to repair of the endometrium at menstruation
Source: eLife. 2022 Dec 16;11:e77663. doi: 10.7554/eLife.77663 (PMC9873258; doi:10.7554/eLife.77663)
Supplement: Supplementary file 1. — (a) Primary and secondary antibodies with associated working dilutions used to detect proteins in mouse uterine tissues. (b) Flow cytometry antibodies selected and optimised to interrogate mesenchymal cell populations in murine uterus. [file elife-77663-supp1.docx]

**Supplementary File 1a.**

| **Antibody** | **Supplier and Cat. No. (concentration)** | **Dilution in**  **NGS** |
| --- | --- | --- |
| **Primary antibodies** |  |  |
| Monoclonal rabbit anti-  PDGFRβ | Abcam, ab32570 (0.15 mg/ml) | 1/1000 |
| Monoclonal rabbit anti-  CD146 | Abcam, ab75769 (0.123mg/ml) | 1/1000 |
| Polyclonal rabbit anti-NG2 | Abcam, ab12905 | 1/600 |
| Polyclonal rabbit anti-CD31 | Abcam, ab28364 (1mg/ml) | 1/500 |
| Polyclonal rabbit anti-  EpCAM | Abcam, ab71916 (1mg/ml) | 1/2000 |
| Polyclonal rabbit anti-  PDGFRA | Santa Cruz Biotechnology, sc-338 (100μg/ml) | 1/1000 |
| **Secondary antibodies** |  |  |
| Goat F(ab) anti-rabbit IgG  H&L (HRP) | Abcam, ab7171 (1mg/ml) | 1/500 |

**Supplementary File 1b.**

| **Antibody** | **Supplier Cat. No. (concentration)** | **Excitation λ (nm)** | **Emission λ (nm)** | **Dilution** |
| --- | --- | --- | --- | --- |
| BV421 anti-mouse CD31 | BioLegend 102423 (0.2mg/ml) | 405 | 421 | 1/200 |
| BV421 anti-mouse CD45 | Fischer Scientific  BDB560501 (0.2mg/ml) | 404 | 448 | 1/100 |
| APC anti-mouse CD146 | BioLegend 134712 (0.2mg/ml) | 650 | 660 | 1/200 |
| BV605 anti-mouse  EpCAM | BioLegend 118227 (0.2mg/ml) | 405 | 603 | 1/400 |
| FITC anti-mouse  CD90 | Invitrogen 11-0902-82 (0.5 mg/ml) | 490 | 525 | 1/100 |
| DAPI (4’,6Diamidino-2Phenylindole,Dilac  tate) | Fischer Scientific D3571 (10mg powder reconstituted to a 0.1μg/μl working solution) | 358 | 461 | 1/1000 |
